# Alain Chippaux nous a quitté le 14 mai 2022 à l'âge de 94 ans

**DOI:** 10.48327/mtsi.v4i2.2024.503

**Published:** 2024-05-14

**Authors:** Jean-Philippe CHIPPAUX, Jean-Paul BOUTIN, Michel DEVELOUX, Alain EPELBOIN, Pierre GAZIN, François MOUTOU, Jean-François PAYS, Eric PICHARD

**Affiliations:** SFMTSI. Hôpital Pitié-Salpêtrière - Pavillon Laveran, 47-83 Boulevard de l’Hôpital, 75013 Paris, France

Site web : http://revuemtsi.societe-mtsi.fr/

**Figure 1:**
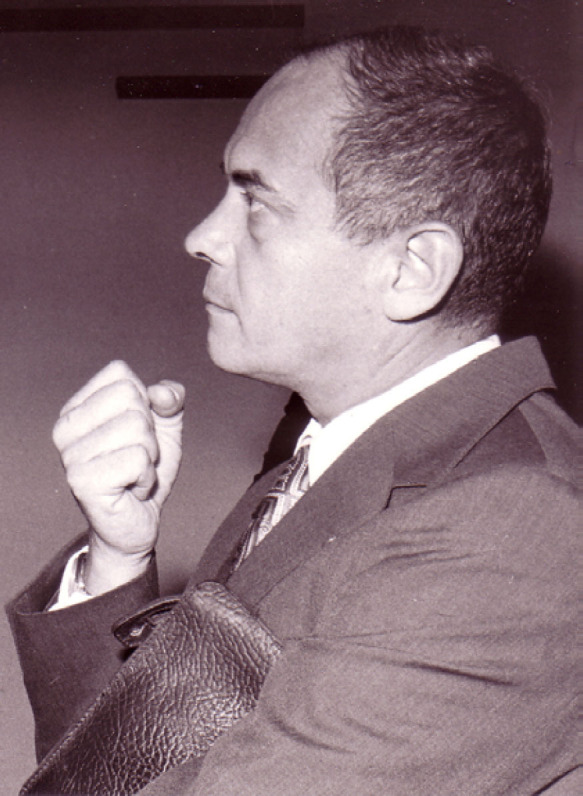
Alain Chippaux en 1977 à Abidjan, archive familiale Alain Chippaux in 1977, Abidjan, family archive

Il y a deux ans disparaissait Alain Chippaux, membre éminent de notre Société.

**Figure 2:**
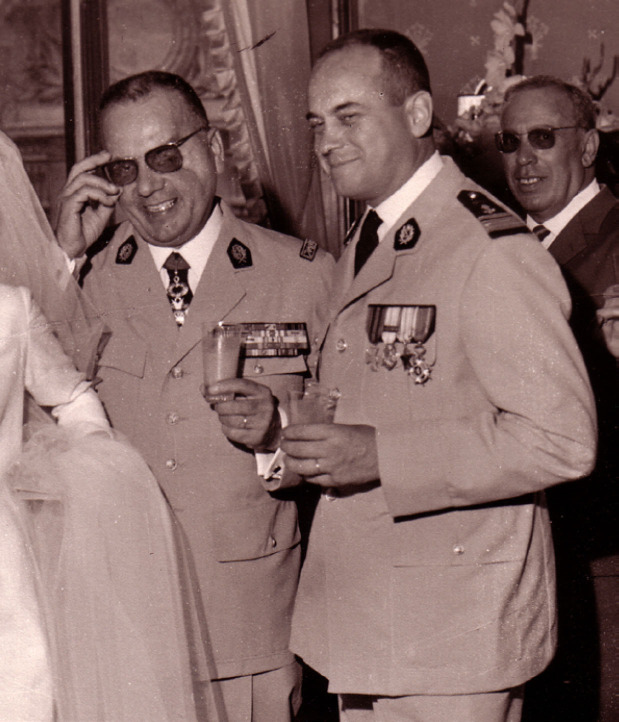
Alain Chippaux (à droite), avec son frère Claude (à gauche), en 1968 à l'École du Pharo, Marseille, archive familiale Alain Chippaux (right), with his brother Claude (left), in 1968 at the École du Pharo, Marseille, family archive

Né le 28 février 1928 à Paris, il a été marqué dans sa jeunesse par la disparition de son père en 1939 – il venait d’avoir 11 ans – et par la guerre. À 16 ans, combattant volontaire de la résistance dans le maquis du Haut-Ognon du département de Haute-Saône, il fut blessé par balle au cours d’une mission de liaison le 21 septembre 1944. Le lendemain, le 22 septembre 1944, son frère Claude^1^1Claude Chippaux fit une brillante carrière. En 1954, il organisa l’évacuation des blessés de Diên Biên Phu. Chirurgien, anatomiste et anthropologue physique, il étudia les mutilations volontaires, notamment le petit pied de la chinoise. Il dirigea le Pharo de 1967 à sa retraite en 1971. Président des Gueules Cassées de 1974 à sa mort en 1984, il contribua à la création et au développement du Loto (Fig. 2) de 19 ans son aîné, médecin capitaine appartenant au même maquis, a lui-même été très grièvement blessé à la face.

Soigné en Suisse, Alain Chippaux en revint quelques semaines plus tard, déguisé en fille pour échapper à la vigilance de l’occupant.

Après son baccalauréat, il fut élève de l’école annexe de médecine navale de Rochefort (1948-1949) avant d’intégrer la Faculté de médecine de Lyon comme élève de l’École du service de santé militaire. Il soutint sa thèse en 1954 et rejoignit l’École d’application du service de santé des troupes coloniales au Pharo à Marseille.

À la sortie du Pharo, il fut affecté comme médecin-chef du secteur n° 1 du Service commun de lutte contre les grandes endémies au Congo Brazzaville d’octobre 1955 à septembre 1958. Pendant ces trois années, il y supervisa les interventions de lutte contre la maladie du sommeil qui décimait l’Afrique depuis le début du XX^e^ siècle.

Dès son retour en France, il passe avec succès le concours d’assistanat de biologie des troupes de Marine qui lui ouvre le « Grand Cours » de microbiologie à l’Institut Pasteur à Paris, d’octobre 1959 à juillet 1960.

**Figure 3:**
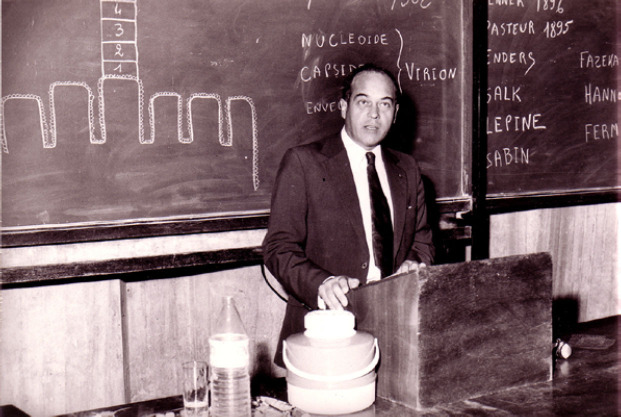
Alain Chippaux, à la Faculté de médecine d'Abidjan en 1972, archive familiale Alain Chippaux, at the Abidjan Faculty of Medicine in 1972, family archive

À l’issue du Cours, la direction du nouvel Institut Pasteur de Bangui (République centrafricaine), essentiellement dédié à l’étude des arbovirus, lui est confiée. Il y met en place notamment la surveillance des virus de la fièvre jaune, Chikungunya et Zika parmi de nombreuses autres maladies virales. Son épouse, Claude Chippaux-Hyppolite, médecin et diplômée du cours de l’Institut Pasteur la même année que lui, le secondera de 1960 à 1966. Cette dernière, après leur séjour à Bangui, a fait son propre chemin, d’abord à la Faculté de médecine de Marseille (1967-1973), puis à celles d’Abidjan (1973-1979) (Fig. 3 et 4) et devint professeur des universités (microbiologie) successivement à Nantes, puis à Reims. Reçu au concours de la spécialité des hôpitaux des armées en microbiologie, option tropicale, en 1966, Alain Chippaux est affecté au Pharo jusqu’en 1971 comme chef du laboratoire de recherches en virologie et épidémiologie appliquées aux arbovirus.

**Figure 4:**
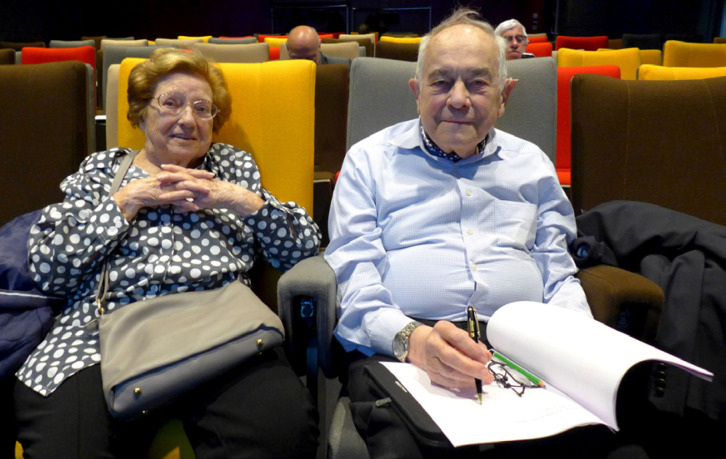
Claude et Alain Chippaux le 29 mai 2019 à la Journée de la SPE « Rongeurs en pathologie exotique » à l'Institut Pasteur (crédit photo : Alain Epelboin) Claude and Alain Chippaux on May 29, 2019 at the SPE Day “Rodents in exotic pathology” at the Institut Pasteur (photo credit: Alain Epelboin)

Il étudiera les arbovirus de Camargue et du littoral méditerranéen, notamment le virus West-Nile récemment isolé de chevaux et d’humains.

En 1971, il prend, la direction du nouvel Institut Pasteur de Côte d’Ivoire à Abidjan dont la virologie était l’objectif initial essentiel. En 1979, il rentre à Paris qu’il ne quittera plus. Sa grande expérience de la virologie et des arbovirus en particulier lui permet d’être nommé directeur du département de contrôle des vaccins viraux et des produits dérivés du sang au Laboratoire national de la santé, service auquel était rattaché le Centre de référence du virus variolique et autres *Orthopoxvirus.* Il occupa cette fonction d’août 1979 à février 1988. C’est à ce titre que, dès mai 1983, il alerta le directeur général de la santé sur le risque de transmission du sida chez les receveurs de produits sanguins administrés sans inactivation virale, plaidant pour accélérer sa mise en place afin de réduire au maximum le risque d’apparition du sida chez les hémophiles et les personnes transfusées. Médecin-chef des services hors classe, grade ultime dans la hiérarchie, il prend alors sa retraite du Service de santé des armées. Il est immédiatement recruté par l’Institut Pasteur de Paris pour diriger jusqu’en avril 1994, le Laboratoire des arbovirus qui est également Centre national de référence et Centre collaborateur de l’OMS.

La retraite d’Alain Chippaux sera très active jusqu’à pratiquement la fin de sa vie. Il sera membre du Comité technique des vaccinations, de la Commission de réparation des accidents vaccinaux et de la Commission d’autorisation des médicaments. Il effectuera des missions en Afrique occidentale et centrale pour l’Organisation des Nations unies pour le développement industriel (ONUDI) afin d’étudier les possibilités d’installation de laboratoires de contrôle des vaccins et sérums à usage humain sur le continent africain.

Toute sa vie, il s’est impliqué dans de nombreuses associations, notamment les Scouts de France dont il fut délégué aux relations internationales. Il fut un membre majeur de l’Association des anciens élèves de l’Institut Pasteur. Membre du Conseil d’administration depuis 1983, il en fut, de 1990 à 2015, le secrétaire général. Il fut également archiviste de l’Association (1984-2015) et responsable de la commission du Bulletin (1995-2000).

Élu membre titulaire de la Société de pathologie exotique (SPE) en 1966, puis au Conseil d’administration en 1986, il a participé aux travaux du Comité de rédaction du *Bulletin* de 1990 à la fin des années 2010. Corédacteur en chef avec Alain Epelboin, il a renforcé et systématisé l’évaluation des manuscrits par les pairs.

À partir de 2004, membre du Comité éditorial, il a relu et corrigé la plupart des articles avant leur envoi à l’éditeur. Représentant de la Fédération européenne des sociétés de médecine tropicale, il en fut le secrétaire dès sa fondation en 1995 jusqu’en 2000, date à laquelle il céda sa place à Pierre Ambroise-Thomas pour se consacrer plus complètement à la SPE. Il a été secrétaire général de la SPE de 1995 à 1999, puis président de 1999 à 2003. Il contribua fortement à l’organisation du IV^e^ congrès de la Fédération européenne de médecine tropicale et santé publique à Marseille en 2005, à l’occasion du centenaire de l’École du Pharo. Alain Chippaux est l’auteur de nombreux articles et ouvrages sur les arbovirus, les vaccins et les vaccinations contre les maladies à virus, la santé publique et les bonnes pratiques de sécurité au laboratoire.

Il était officier de la Légion d’honneur (1987), officier de l’ordre national du Mérite (1982), titulaire de la Croix du combattant volontaire de la Résistance, de la médaille d’Outre-Mer (1970), de la médaille d’honneur (argent) du Service de santé des armées (1985) et chevalier de l’ordre du Mérite centrafricain.

Alain Chippaux laisse le souvenir d’un homme d’une grande compétence, plutôt modeste, discret, d’humeur égale et toujours chaleureux.

**Figure 5:**
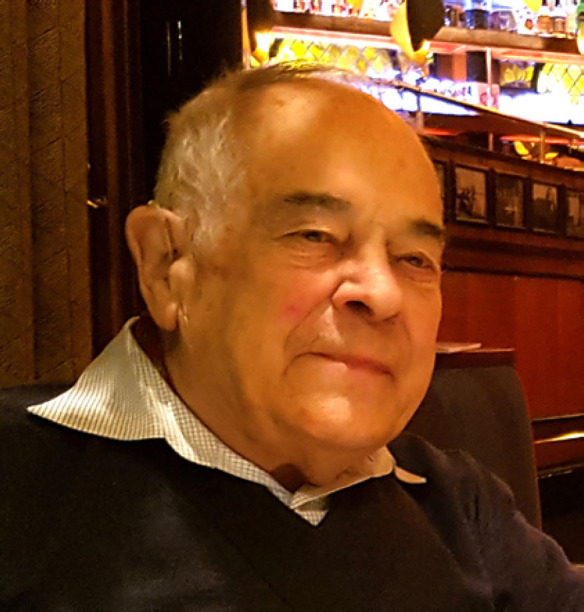
Alain Chippaux, octobre 2019, archive familiale Alain Chippaux, October 2019, family archive
